# Supplementary material for: Predicting visual function by interpreting a neuronal wiring diagram
Source: Nature. 2024 Oct 2;634(8032):113–23. doi: 10.1038/s41586-024-07953-5 (PMC11446822; doi:10.1038/s41586-024-07953-5)
Supplement: Supplementary file 6 — CRF and ERF predictions for individual TmY4 and TmY9 cells. Analogous to Supplementary Data 3, but for TmY target types. Shown are the top four monosynaptic pathways, the strongest pathway passing through each of the top ten intermediary types (ranking from Extended Data Fig. 7), and the trisynaptic pathway Tm1–TmY–Dm3–TmY (see the section entitled Prediction of spatial normalization). [file 41586_2024_7953_MOESM6_ESM.zip › DataS4/TmY9qΓèÑ/720575940635271007.pdf]

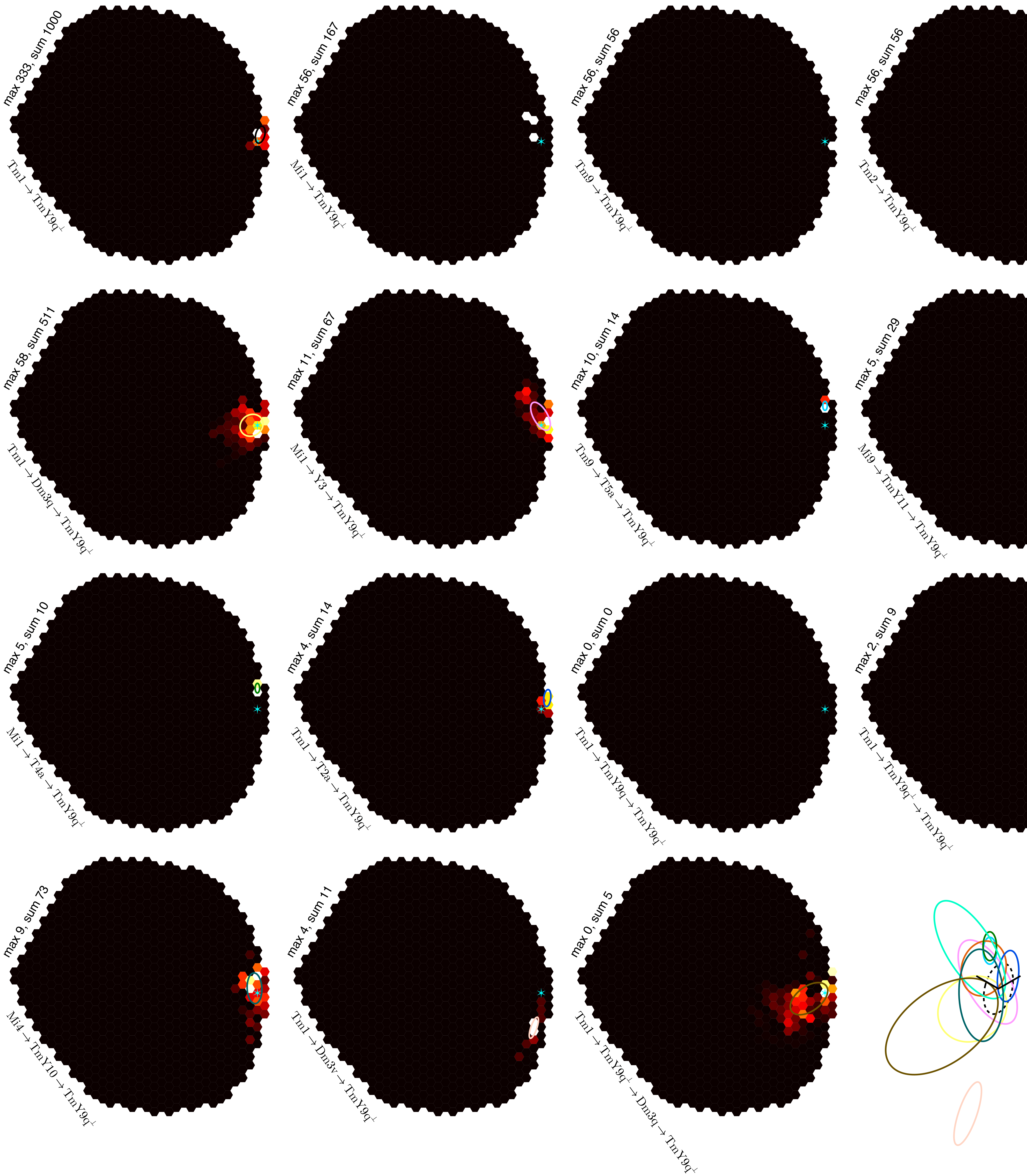
max 333, sum 1000
Tm1 → TmY9q$^{\perp}$
max 56, sum 167
Mi1 → TmY9q$^{\perp}$
max 56, sum 56
Tm9 → TmY9q$^{\perp}$
max 56, sum 56
Tm2 → TmY9q$^{\perp}$
max 58, sum 511
Tm1 → Dm3q → TmY9q$^{\perp}$
max 11, sum 67
Mi1 → Y3 → TmY9q$^{\perp}$
max 10, sum 14
Tm9 → T5a → TmY9q$^{\perp}$
max 5, sum 29
Mi9 → TmY11 → TmY9q$^{\perp}$
max 5, sum 10
Mi1 → T4a → TmY9q$^{\perp}$
max 4, sum 14
Tm1 → T2a → TmY9q$^{\perp}$
max 0, sum 0
Tm1 → TmY9q → TmY9q$^{\perp}$
max 2, sum 9
Tm1 → TmY9q$^{\perp}$ → TmY9q$^{\perp}$
max 9, sum 73
Mi4 → TmY10 → TmY9q$^{\perp}$
max 4, sum 11
Tm1 → Dm3v → TmY9q$^{\perp}$
max 0, sum 5
Tm1 → TmY9q$^{\perp}$ → Dm3q → TmY9q$^{\perp}$
